# Bipartite Anterior Extraperitoneal Teratoma: Evidence for the Embryological Origins of Teratomas?

**DOI:** 10.1155/2011/208940

**Published:** 2011-08-28

**Authors:** D. J. B. Keene, R. J. Craigie, A. Shabani, G. Batra, S. Hennayake

**Affiliations:** ^1^Department of Paediatric Surgery, Royal Manchester Children's Hospital, Oxford Road, Manchester, M13 9WL, UK; ^2^Department of Paediatric Radiology, Royal Manchester Children's Hospital, Oxford Road, Manchester, M13 9WL, UK; ^3^Department of Paediatric Pathology, Royal Manchester Children's Hospital, Oxford Road, Manchester, M13 9WL, UK; ^4^Department of Paediatric Urology, Royal Manchester Children's Hospital, Oxford Road, Manchester, M13 9WL, UK

## Abstract

Teratomas are thought to arise from totipotent primordial germ cells (PGCs) Dehner (1983) which may miss their target destination Moore and Persaud (1984). Teratomas can occur anywhere from the brain to the coccygeal area but are usually in the midline close to the embryological position of the gonadal ridges Bale (1984), Nguyen and Laberge (2000). We report a case of a bipartite anterior extraperitoneal teratoma. This is an unusual position for a teratoma, but one which may support the “missed target” theory of embryology.

## 1. Case Report

**Figure 1:**
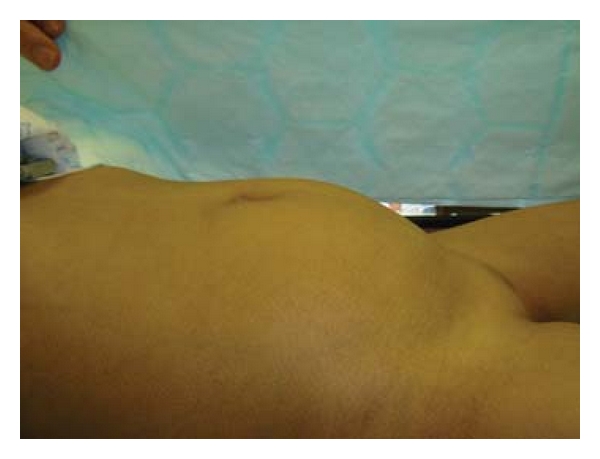
Prominent mass lower abdomen.

A 4-year-old girl was referred with an 18-month history of a lower abdominal swelling (Figure 1). She had lower abdominal swelling associated with intermittent abdominal pains and occasional wetting. No concerns had been raised antenatally. Examination was unremarkable except for a mobile tennis ball sized mass in the lower abdomen.

**Figure 2:**
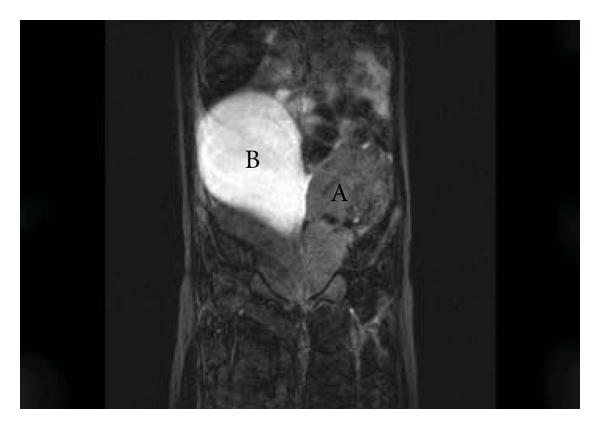
T2 coronal section of fat-suppressed abdominal magnetic resonance scan showing different signal intensities in solid component (A) and high signal in cystic component (B).

**Figure 3:**
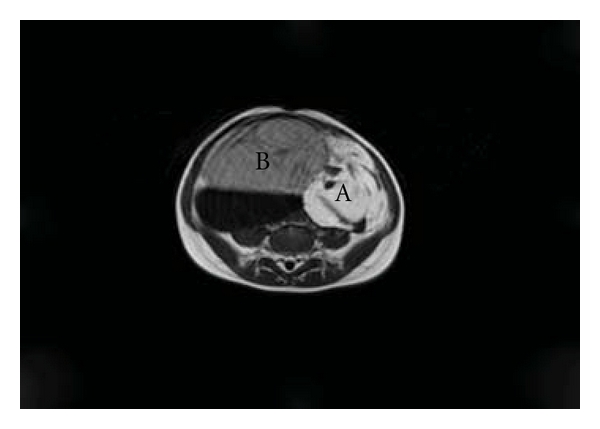
T1 transverse section of abdominal magnetic resonance scan showing intermediate signal in solid component (A) with low signal areas representing calcification and low signal in cystic component (B).

Magnetic resonance imaging demonstrated a 16 × 15 × 12 cm mass with cystic and solid components (Figure 2). The cystic component (a) was anterior and superior to the bladder showing high signal on T2-weighted images (Figure 2). The solid component (b) was posterolateral showing intermediate signal on T1-weighted images with low signal areas representing calcification (Figure 3). These findings resulted in a decision to remove the lesion.

**Figure 4:**
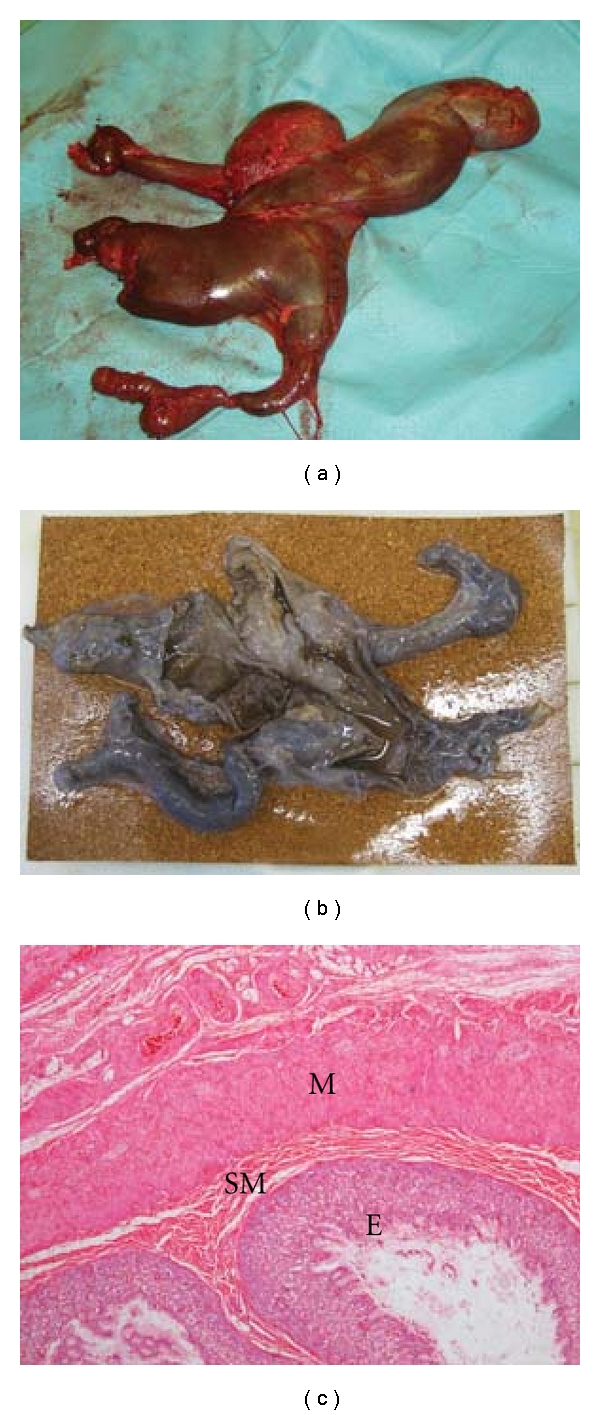
Cystic component of mass. (a) Macroscopic appearance of fresh unfixed specimen. (b) Macroscopic appearance of fixed specimen with cyst opened. (c) Microscopy of haematoxalin and eosin-stained specimen at x2.5 magnification. Layers of enteric lined mucosa comprising of columnar epithelia (E), submucosa (S), and muscularis (M).

**Figure 5:**
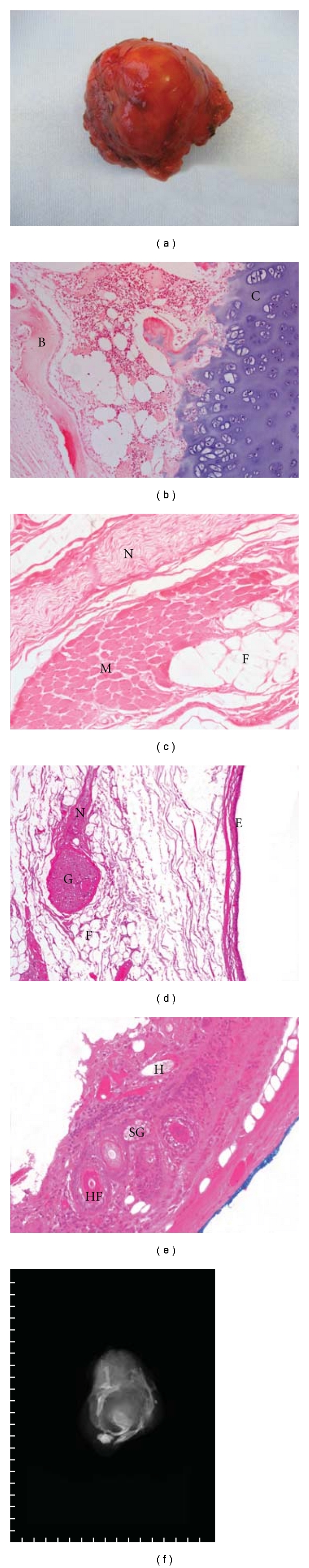
Solid component of mass stained with haematoxalin and eosin. (a) Macroscopic appearance of fresh unfixed specimen. (b) Microscopy x10 magnification. Osteoblast layer on the surface of bone (B) adjacent to cartilage (C). (c) Microscopy x20 magnification. Skeletal muscle fibres (M), nerve (N), and fat (F). (d) Microscopy x10 magnification. Nerve cells (N), ganglion (G), fat (F) and, epithelia lined cyst (E). (e) Microscopy x20 magnification. Pilosebaceous unit comprising of sebaceous gland (SG), hair follicle (HF), hair (H). (f) Plain X-ray of solid component showing skeletal structure.

At surgery a bipartite mass was found. Extraperitoneal and anterior to the bladder was a cystic bowel-like structure which was not continuous with true bowel (Figure 4(a)). Separate from this and also extraperitoneal was a large calcified mass (Figure 5(a)) to the left of the midline abutting the iliac vessels at the level of the pelvic brim. On opening the peritoneum at the end of the procedure healthy bowel, uterus, and both ovaries were present.

Histological examination of the cystic bowel-like component (Figure 4(b)) demonstrated gastrointestinal structure throughout with mucosa, submucosa, muscularis propria, and serosa. The mucosa was mostly of intestinal type in the tissue sampled, but gastric type mucosa was also noted.

The calcified component had appearances consistent with teratoma fat, bone, cartilage, ciliated epithelium, smooth and skeletal muscle, and a cyst containing epidermal appendages lined by giant cells. X-rays showed irregular areas of well-formed bone in a circular formation but no true skeletal formation (Figure 5(f)).

Eighteen months after resection, the child remains well and is asymptomatic. Repeat imaging and tumour markers have not shown any sign of recurrence.

## 2. Discussion

Teratomas are defined as having three embryonic layers, although recent classifications include monodermal types [1]. In our case report, tissues from all three embyronal layers were present: enteric lined mucosa (Figure 4(b)) (endodermal component), bone, cartilage, and muscle (Figures 5(b) and 5(c)) (mesodermal component), and epidermal appendages (Figures 5(d) and 5(e)) (ectodermal component).

**Figure 6:**
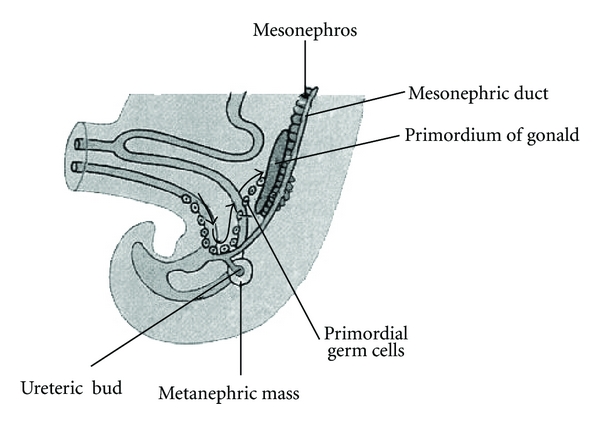
Migrational theory of primordial germ cells [4]. Reproduced by permission of Elsevier Saunders.

This case report identifies a bipartite teratoma in an unusual position. There are 3 theories for the origin of teratomas [2]. The first theory is that teratomas arise from remnants of the primitive streak or primitive node. During week 3 of development, midline cells at the caudal end of the embryo divide rapidly by the process of “gastrulation” giving rise to all the three germ layers of the embryo [3]. By the end of week 3, the primitive streak shortens and disappears. The second theory is that teratomas originate from totipotent primordial germ cells (PGCs) [1]. These cells develop among the endodermal cells of the yolk sac near the origin of the allantois and migrate to the gonadal ridges during weeks 4 and 5 of gestation (Figure 6). Some cells may miss their target destination and produce a teratoma [4]. The third theory is incomplete twinning, which has received less support recently [1, 5, 6].

Primordial germ cells lose their potency, as gastrulation progresses, which is associated with spatial restriction [7]. Sacrococcygeal and pelvic locations for teratomas predominate [8] which fit well with gastrulation theory, however this fails to explain how teratomas develop at distant sites with respect to the primitive streak. Primordial germ cells are known to migrate from the allantois to the gonadal ridges (Figure 6) and this migration pathway corresponds strikingly well to the anterior extraperitoneal location in this patient. Once embryonic stem cells are in an ectopic microenvironment, they are known to have the potential to develop into teratomas [9]. A failure of migration would explain the unusual location of the bipartite teratoma in this case and also support the “missed target hypothesis.”

In mammals, the PGCs are observed in an extraembryonic region near the yolk sac, they translocate to the endodermal epithelium of the hindgut, separate from the gut epithelium to enter the dorsal mesentery, and finally migrate to form the gonadal ridge [2]. There are different mechanisms proposed for guiding this migration including pseudopodial structures, chemotactic factors, basement membrane or extracellular molecules, and interaction between PGCs to explain the migratory pathway [10, 11]. If the missed target theory is correct, an interruption has occurred in the migration of totipotent germ cells which must have occurred early in embryogenesis, prior to week 5 of gestation. The size of the tumour at resection and the absence of findings at antenatal imaging demonstrate that although initial development was early in gestation, the growth of this teratoma was primarily a postnatal event.

The histology in this case showed teratomatous features some of which are present in fetus in fetu. For a diagnosis of fetus in fetu, the mass should include vertebrae or notochord and a high degree of structural organisation [12–14]. Plain X-rays of part of the mass showed irregular areas of well-formed bone (Figure 5(f)), but no evidence of the vertebrae thereby confirming that the diagnosis is a teratoma.

## 3. Conclusion

This case report questions the validity of the gastrulation theory of teratoma formation. The bipartite nature of the tumour and position along the migratory pathway of primordial germ cells, support the missed target theory for teratoma formation.
